# Not just fillers: the emerging role of bone marrow adipocytes in bone and bone marrow homeostasis

**DOI:** 10.1242/bio.062461

**Published:** 2026-09-22

**Authors:** Giorgia Farinacci, Biagio Palmisano

**Affiliations:** ^1^ Department of Molecular Medicine, Sapienza University of Rome, Rome 00161, Italy; ^2^ Institute for Complex Systems, National Research Council of Italy, Rome 00185, Italy

**Keywords:** Bone marrow adipose tissue (BMAT), MALPs, Skeletal stem cells, Lipolysis, Bone remodeling, Hematopoietic niche, Osteoporosis

## Abstract

Bone marrow adipose tissue (BMAT) is a phylogenetically conserved fat depot occupying a substantial fraction of the medullary cavity in vertebrates, yet its functional significance has only recently begun to be appreciated. Long dismissed as an inert filler, BMAT is now recognized as a dynamic and metabolically active compartment participating in the regulation of bone remodeling and hematopoiesis. Bone marrow adipocytes (BMAds) originate from skeletal stem cells (SSCs) and are anatomically and functionally distinct from peripheral white adipocytes, exhibiting a unique lipid metabolism and reduced lipolytic responsiveness under basal conditions. Two spatially and functionally distinct BMAT subtypes, i.e. regulated (rBMAT) and constitutive (cBMAT), respond differently to physiological and pathological stimuli, including caloric restriction, cold exposure, irradiation, and disease.

BMAT expands in conditions such as osteoporosis and aging, while contracting in the context of bone metastasis and hematological malignancies, underscoring its tight coupling to the marrow microenvironment. Mechanistically, BMAT regulates bone remodeling by expressing osteoclastogenic factors and through lipid-mediated paracrine signaling, and supports or restrains hematopoietic stem cells, depending on context and skeletal site, by secreting key molecules in the hematopoietic niche. Emerging evidence from genome-wide Mendelian randomization studies now establishes a causal link between elevated BMAT and osteoporosis risk in humans. In the oncological setting, BMAds fuel tumor cell metabolism, promote cancer cell migration and chemoresistance, and remodel the marrow niche to favor malignant progression.

This article critically reviews the current understanding of BMAT biology, from its anatomical organization and developmental origins to its roles in skeletal and hematopoietic homeostasis.

## Teaser

**Summary:** Our bones contain fat. This is known as bone marrow adipose tissue, and it is not an inert tissue, but it is able to influence bone and bone marrow homeostasis.

## INTRODUCTION

Our bones contain fat. While this notion is very well known among butchers and whichever restaurateur has ever cooked a delicious dish from Italian cuisine (‘ossobuco alla milanese’), it still surprises most students and researchers when they first encounter it. Why does bone marrow have fat? This Review will attempt to address this question. We are, perhaps somewhat opportunistically, sidestepping an equally important prior question – whether bones need fat at all. The evolutionary conservation of bone marrow adiposity across vertebrates suggests they do (Craft and Scheller, 2017), yet the direct experimental evidence remains insufficient to settle the matter definitively. We therefore leave this question open and focus instead on the functional question of why.

As with any cell type, tissue or organ in our body, adipocytes in the bone marrow are there for a reason and their tight control seen in both physiological and pathological conditions explains the need of the bone marrow to regulate these lipid-laden cells.

Bone marrow adipocytes are anatomically part of the bone marrow stroma. The etymology of the word stroma – from the Greek ‘στρῶμα’, meaning ‘layer’, ‘bed’, ‘bedcovering’, or ‘mattress’ – already suggests one fundamental function for bone marrow adipocytes (BMAds) and the other stromal cells: support of the parenchyma, which in the bone marrow is represented by the hematopoietic tissue.

In the early 1970s, Tavassoli was the first describing BMAds ultrastructure, highlighting the morphological differences in organelle number and distribution compared to extramedullary adipocytes, and precursor from which they originate (Tavassoli, 1976). Interestingly, without the need of mouse genetics, lineage tracing and advanced microscopy techniques, he was able to describe that the cell giving rise to marrow adipocytes was completely different from the one originating from peripheral white adipose tissues, as it did not contain glycogen, was not abundant in endoplasmic reticulum and did not produce collagen (Tavassoli, 1976). Since then, the bone marrow adipose tissue (BMAT) field has received enormous attention, especially in the last 15 years, with seminal studies that have recognized it as an important endocrine organ possibly contributing to systemic metabolism. This Review will, however, put a lens on the role of BMAT in bone and marrow homeostasis, highlighting recent insights that provide us with a more comprehensive understanding of this adipose tissue depot.

## Anatomy of BMAT

In healthy humans at the peak of their bone mass (typically by the age of 25), fat accounts for more than 70% of bone marrow volume (Fig. 1A). Based on the amount of BMAds that are present within the medullary cavity, we distinguish between two different forms of bone marrow, namely the red and yellow bone marrow. The ‘red marrow’ is a hematopoietically active marrow, able to produce blood cells, while the ‘yellow marrow’ is on the contrary considered to be hematopoietically inactive. The red marrow contains isolated, sparse adipocytes among the hematopoietic cells, while the yellow marrow is composed predominantly of fat cells that are larger than those in the red marrow and form a compact adipose tissue that is morphologically very similar to peripheral white adipose tissues (Fig. 1B,C). In the yellow marrow, cells from the hematopoietic lineage are poorly represented (Fig. 1C). In adults, yellow marrow constitutes half of the total bone marrow weight, while red marrow accounts for the other half. However, red marrow composes 50% of adipose tissue, therefore, approximately 75% of adult bone marrow is composed by fat cells. The two marrow types also differ based on their chemical composition, with the red marrow containing more water and more protein than the yellow, but less fat (Steiner et al., 1993; Vogler and Murphy, 1988) (Fig. 1D). As for the vascular supply, the predominantly sinusoidal system of the red marrow is replaced in the yellow marrow by capillaries, venules and veins with thin walls. This conversion likely reflects multiple concurrent changes: the loss of the hematopoietic demand for diapedesis (the process by which blood cells migrate across the sinusoidal wall into the bloodstream), which renders the specialized sinusoidal architecture unnecessary; the influence of adipocytes, which may secrete factors regulating the local vasculature; and, possibly, the direct mechanical pressure exerted by these bulky cells on the surrounding vessel walls.

**Fig. 1.**
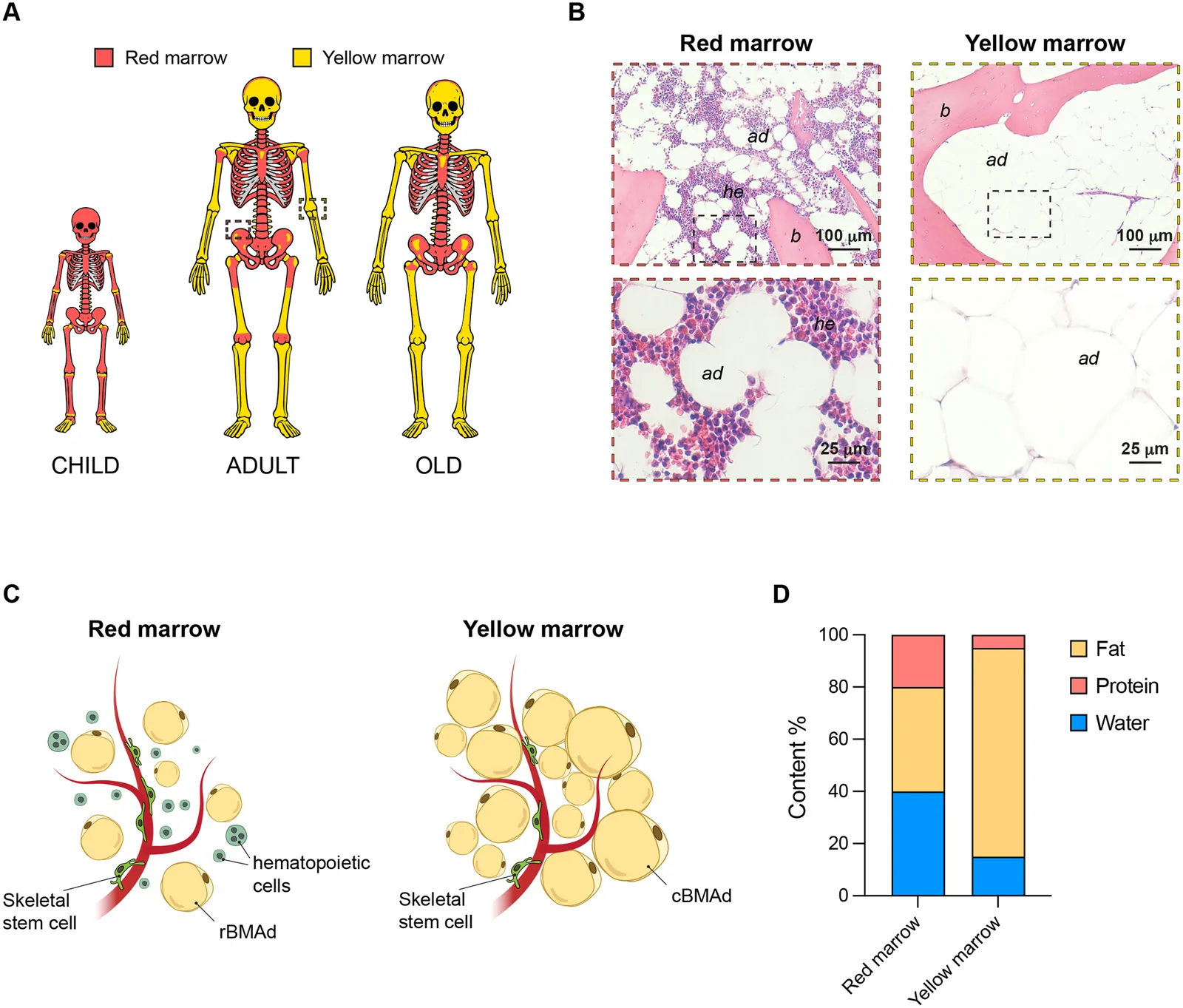
**Anatomy of bone marrow adipose tissue.** (A) Schematic representation of red and yellow marrow distribution in human skeleton in child, adult and old. The skeleton sketches were generated using ChatGPT, vectorized and then modified with Adobe Illustrator. (B) Hematoxylin and Eosin-stained tissue sections of human bone biopsies of skeletal sites containing red marrow (iliac crest, male donor, 32 years old) and yellow marrow (elbow, male donor, 45 years old), obtained as surgical waste from patients undergoing corrective surgeries upon Local Ethical Committee (Comitato Etico Territoriale Lazio Area 1) approval. b, bone; ad, bone marrow adipocyte; he, hematopoietic cells. (C) Schematic representation of the cellular composition of red marrow, containing sparse regulated bone marrow adipocytes (rBMAd) among the hematopoietic cells, and yellow marrow, which is composed mainly of large constitutive bone marrow adipocytes (cBMAd). (D) Chemical composition of red marrow containing 40% water, 40% fat and 20% protein, and yellow marrow composed of 15% water, 80% fat and 5% protein (Steiner et al., 1993; Vogler and Murphy, 1988).

The sites of red and yellow marrow are not already established at birth; indeed, the entirety of fetal bone marrow is red, i.e. hematopoietic. A conversion from red to yellow marrow occurs during growth with a predictable centripetal pattern (Emery and Follett, 1964) (Fig. 1A) until 25 years of age, when the conversion is complete and the adult distribution pattern of red versus yellow marrow is largely achieved. The extremities (phalanges of hand and feet) are the first to be filled with BMAT (even before birth; Emery and Follett, 1964) and then the conversion goes from the appendicular toward the axial skeleton. Within the long bones, the conversion occurs from diaphyseal to metaphyseal regions albeit this is not completely homogeneous and may vary at different bone segments, between sexes and during adulthood (Wilson et al., 1996). For instance, females – who have overall less bone marrow fat than males (Xu et al., 2025b) – show more red marrow in the knee region than males, and, paradoxically given the expected age-related red-to-yellow conversion, more red marrow in the distal femur in the 30–60 year age range than in women under 30 (Wilson et al., 1996; Xu et al., 2025a) (Fig. 1A). Some skeletal regions, such as apophyses and epiphyses, are immediately filled with fat after secondary ossification, and are therefore considered yellow marrow (Kricun, 1985; Person et al., 2021). The distribution of red and yellow marrow can vary among individuals and is influenced by sex, age and health status. Nonetheless, overall, an adult pattern presents red marrow in the axial skeleton and in proximal regions of the appendicular skeleton, while yellow marrow fills the rest of the skeleton. After 25 years of age, conversion from red to yellow marrow continues and this is evident in aged individuals, in which loss of trabecular bone is associated with even more increases in BMAT (Xu et al., 2025a) (Fig. 1A).

The precise mechanisms by which this conversion occurs, and its evolutionary reason are largely unknown, although differences in temperature, vascularity and oxygen tension among different skeletal sites and especially between appendicular and axial bone have been implicated as potential factors in the phenomenon (Vogler and Murphy, 1988).

While the adipocytes in red and yellow marrow differ from a morphological point of view, with those in red marrow being smaller in size than the ones in yellow marrow (Scheller et al., 2015) (Fig. 1B,C), it is not known if they have different functions, at least in humans. Insights into the functions and the different regulation of the two types of BMAT have been provided by animal studies. Tavassoli was the first to describe the distinction between subtypes of BMAT (Tavassoli et al., 1974), observing that based on the response to hematopoietic expansion there were two populations of bone marrow adipose cells, the one in red marrow that was ‘labile’, as it could be resorbed, and the one in the yellow marrow that was ‘stable’, as it was not altered by the expansion of the hematopoietic tissue. These two BMAd subpopulations were then termed ‘regulated’ (rBMAd) and constitutive (cBMAd) (Scheller et al., 2015). rBMAT was defined based on its ability to easily respond to stimuli such as cold exposure or fasting, and its accumulation with age, while cBMAT appeared to be more resistant to those kinds of stimuli (Scheller et al., 2019, 2015). Although the concept of two BMAT subtypes – rBMAT and cBMAT – still stands, a recent study has highlighted site-specific differences within rBMAT and cBMAT regions, raising the question of whether rBMAds or cBMAds from different locations may respond differently to physiopathological stimuli, and therefore whether more than two subtypes of BMAT actually exist (Chen et al., 2026).

## BMAT ontogeny

BMAds originate from multipotent precursor cells with stemness capacity, located in the bone marrow stroma and more precisely wrapping the marrow blood vessels (Fig. 1C). These cells with reticular morphology give rise to the cells of the skeletal lineage, i.e. osteoblasts, fibroblasts, chondroblasts (albeit cartilage generation is rare in postnatal life) and BMAds, and are defined as skeletal stem cells (SSCs). The intense research on SSCs over the last 20 years, using lineage tracing and single-cell RNA sequencing, has allowed us to delineate clear SSC lineage trajectories, distinguishing more adipogenic perivascular SSCs from more chondrogenic osteochondral SSCs, and to identify different levels of commitment (hence the terminology ‘skeletal stem/progenitor cells’, SSPCs) (Ambrosi et al., 2019; Donsante et al., 2021). However, while these studies have helped in identifying cells in humans and mice with more adipogenic or osteogenic capability (Baccin et al., 2020; Bandyopadhyay et al., 2024; Jeffery et al., 2022; Zhong et al., 2020), none of them have elucidated the plausible difference between regulated and constitutive BMAd precursors, which remains, therefore, an unexplored topic. As already extensively reviewed elsewhere (Tencerova et al., 2025), some skeletal segments such as tibiae in rodents are known to contain both cBMAT and rBMAT. The former occupies the distal metaphysis and appears within the first week of age in the mouse, independently of sex; the latter forms in the tibial proximal metaphysis with age, in females earlier than males, and remains highly ‘adjustable’. Why this segment would need both types of BMAds is not known, but it is likely that distinct SSCs, or progenitors, exist for the two marrow locations, as well as for different skeletal sites, as suggested by their distinct responses to pathophysiological stimuli (Chen et al., 2026; Scheller et al., 2019). Evidence for the hypothesis of distinct SSCs across different BMAT compartments comes from *in vivo* data showing that, albeit *Adipoq*-targeted SSPCs can be isolated from both long bones and caudal vertebrae, this population differs between the two skeletal sites, generating osteoblasts and BMAds in the former, while only BMAds in the latter (Palmisano et al., 2022). Studies aiming to identify the precise identity of BMAd progenitors across the different locations are needed.

## More than a ‘filler’ tissue in bone marrow

BMAT was initially thought to be only an inert filler of the bone cavity; however, a lot of evidence has emerged showing that BMAT plays important roles in bone and bone marrow homeostasis. Important processes taking place in the skeleton, such as bone formation and hematopoiesis, require huge amounts of energy to take place and BMAT provides energy support to cells involved in these processes through lipid metabolism. In fact, human BMAds, like white adipocytes, store energy in a single large lipid droplet full of neutral lipids (Attané et al., 2020). Similarly, mouse BMAds store lipids in a large central vacuole and in multiple smaller lipid droplets located at the periphery of the cell indicating an active lipid metabolism (Robles et al., 2019). Moreover, these lipid droplets show a polarization towards the hematopoietic microenvironment with BMAds interacting directly with erythroblast islands and cells of the myeloid/granulocyte lineage and with active osteoblasts, suggesting that BMAds provide energy to these cells through lipid metabolism (Robles et al., 2019).

### To be or not to be a lipolytic adipose tissue in the bone marrow

Lipolysis is a metabolic process through which triacylglycerols stored in lipid droplets inside adipocytes are broken down into glycerol and free fatty acids (FFAs) that are released and used by cells as a source of energy. Lipolysis in BMAds occurs at very low rate – if any – at physiological conditions, or in the absence of catecholamines and other conventional lipolytic stimuli. In humans, BMAds isolated from femoral proximal metaphysis and diaphysis, therefore from both red and yellow marrow sites, show a defective lipolysis with reduced expression of specific enzymes, in particular monoacylglycerol lipase (MGLL), compared to subcutaneous white adipocytes (Attané et al., 2020). More precisely, a recent study (Hernandez et al., 2025 preprint) reported that human rBMAds isolated from the hip carry out a non-canonical lipolysis, involving triglyceride lipase (ATGL), where FFA release from triglycerides is uncoupled from glycerol liberation. Similarly, BMAds in rats and mice have a reduced lipolytic response to β-adrenergic stimuli compared to white adipocytes. In particular, β-adrenergic stimuli induce BMAd lipolysis in proximal skeletal regions rich in rBMAT such as proximal tibia, while distal skeletal regions rich in cBMAT, such as distal tibia and caudal vertebrae, show resistance to β-adrenergic-induced BMAd lipolysis (Scheller et al., 2019). Lipolytic resistance of cBMAT can be overcome by intracerebroventricular infusion of leptin, leading to hypoinsulinemia and hypoglycemia that contribute to the activation of lipolysis and then depletion of cBMAT through catecholamine-independent pathways (Zhang et al., 2026). The absence of sustained lipolytic activity may suggest that this process could be not needed in bone and bone marrow homeostasis. However, a study in which ATGL (*Pnpla2* gene), a rate-limiting lipolytic enzyme, was knocked-out specifically in BMAds [using a *FAC-Cre* mouse model (Li et al., 2022b), hereafter BMAd-Cre], highlighted that although BMAd lipolysis is not fundamental for bone homeostasis in basal conditions, it becomes important in conditions of reduced energy supply (Li et al., 2022b). In particular, BMAd lipolysis is fundamental under caloric restriction to maintain bone mass and myelopoiesis, during bone regeneration for the healing process and for maintaining bone mass during cold exposure (Li et al., 2022b). On the same line, BMAT lipolysis is enhanced in conditions characterized by a high bone remodeling rate such as human hand osteoarthritis, in which BMAd lipolysis may contribute to subchondral bone formation since the enhanced osteoblast activity is associated with an increased expression of lipolytic enzymes in BMAds (Maniglio et al., 2025). Therefore, BMAT lipolysis may not be fundamental in basal conditions but becomes important in conditions of high energy demand. Beside lipolysis, BMAds exhibit a cholesterol-oriented lipid metabolism with enhanced expression of proteins and enzymes involved in cholesterol metabolism compared to subcutaneous white adipocytes (Attané et al., 2020). Moreover, both animal and human BMAds have an altered glucose metabolism and decreased insulin responsiveness, with a high basal glucose uptake in human BMAT suggesting that it may influence systemic glucose homeostasis (Suchacki et al., 2020).

### A new player in bone remodeling

It has been shown that BMAds are also able to regulate bone remodeling through the expression of some important osteoclastogenic factors such as RANKL and OPG. *In vitro*, RANKL expression in human primary BMAds can be enhanced by some stimuli such as TNFα and dexamethasone. As a result, BMAds can promote osteoclastogenesis in a RANKL-dependent manner (Goto et al., 2011a,b; Hozumi et al., 2009). Also in mice, BMAT is a source of RANKL. Following specific deletion of parathyroid hormone receptor (PTH1R) in bone marrow stromal cells using *Prx1-Cre* (*Prx1-Cre;PTH1R^fl/fl^* mice), mice develop a low bone mass phenotype associated with an increase in BMAd number, which express RANKL, thereby inducing bone resorption (Fan et al., 2017). While these studies have tried to highlight the potential expression of RANKL by lipid-laden mature BMAds, recent studies have demonstrated that the main contribution to RANKL and M-CSF production by the BMAd lineage may be provided by a population of fat-free BMAd precursor cells, namely the marrow adipogenic lineage precursors (MALPs). These are one of the major sources of RANKL inside bone marrow and stimulate osteoclast maturation through cell–cell interaction (Lu et al., 2025; Yu et al., 2021). In fact, removing RANKL in MALPs using the *Adipoq* promoter (*Adipoq-Cre*;*Tnfsf11^fl/fl^* mice) results in a high bone mass phenotype with reduced osteoclast number and attenuated bone formation, indicating that MALPs strongly contribute to trabecular bone remodeling (Hu et al., 2021; Lu et al., 2025; Yu et al., 2021). Moreover, MALP-derived RANKL also contributes to pathologic bone loss, since its deletion prevents LPS and ovariectomy-induced bone loss (Yu et al., 2021). Tamoxifen-inducible deletion of RANKL in MALPs showed that MALP-derived RANKL is also important for trabecular bone turnover in adult bone homeostasis (Lu et al., 2025). In addition to RANKL, MALPs seem to express high levels of M-CSF placing them among major regulators of bone resorption and remodeling (Inoue et al., 2023; Zhong et al., 2023). However, recent findings have demonstrated that the *Adipoq* promoter is not specific to MALPs or BMAds, as it also targets cells with skeletal stem/progenitor ability, and therefore is capable of generating osteoblasts (Jeffery et al., 2022; Mukohira et al., 2019; Palmisano et al., 2024, 2022). These findings therefore call for caution when interpreting results based on *Adipoq-*driven models (Palmisano et al., 2024). This issue can be, in part, overcome by a newly developed mouse model (*FLPo*-activated *Adiponectin-Cre*, FAC-Cre), generated using a dual-recombinase intersectional strategy – combining *Adipoq* and *Osx* promoter – that restricts genetic recombination exclusively to BMAds, thereby avoiding unwanted targeting of peripheral white/brown adipocytes or osteoblasts (Li et al., 2022b). However, even this model has revealed some leakiness in *Adipoq*-expressing stromal cells (Li et al., 2022b; Palmisano et al., 2024), highlighting the ongoing difficulty of selectively targeting BMAd precursors from the mature cell population.

In addition to the main role in regulating bone resorption, BMAT plays a role in physiological bone formation. In fact, BMAT depletion through expression of diphtheria toxin A or deletion of *Pparg* using *Adipoq-Cre*, or *BMAd-Cre* mice, results in increased bone mass in BMAd-enriched skeletal sites (Li et al., 2022a; Zhang et al., 2021; Zou et al., 2020). This increase in bone mass is observed in regions of rBMAT, such as distal femurs and proximal tibiae (Zhang et al., 2021; Zou et al., 2020), but also in those rich in cBMAT, such as distal tibiae and caudal vertebrae, where cBMAT depletion is followed by an increase in cortical and trabecular bone formation (Li et al., 2022a). Moreover, loss of cBMAT also protects mice from caloric restriction and ovariectomy-induced cortical bone loss and promotes fractured-bone healing processes (Li et al., 2022a). Altogether, these findings suggest that the BMAT lineage, namely MALPs and BMAds, is able to regulate bone remodeling by acting on both resorption and formation and therefore needs to be considered as an important player in this process.

### The intimate relationship with hematopoiesis

As part of the bone marrow stromal compartment, BMAT supports the parenchyma, i.e. the hematopoietic tissue. However, the role of BMAT in regulating hematopoiesis remains controversial, with some reports suggesting that BMAT positively regulates hematopoiesis while others demonstrating the opposite. One way to understand the relationship between BMAT and hematopoiesis is to study the yellow marrow, where BMAT is abundant, but hematopoiesis is scarce or absent. Indeed, hematopoiesis is physiologically minimized in BMAT-rich caudal vertebrae, where hematopoietic stem cells (HSCs) and progenitors are reduced in number compared to other skeletal sites characterized by fewer adipocytes (Naveiras et al., 2009).

In the A-ZIP/F-1 mouse model, a strain lacking white adipose tissue and with reduced brown adipose tissue (Moitra et al., 1998), tail vertebrae display dramatically reduced BMAT and restored hematopoisis comparable to that of thoracic vertebrae (Naveiras et al., 2009). Another informative model for studying the relationship between BMAT and hematopoiesis is myeloablation, in which BMAds become the predominant stromal cell population in the bone marrow (Zhou et al., 2017). Specifically, BMAds accumulate in the bone marrow of wild-type (WT) mice within 2 weeks after sublethal irradiation and subsequently decline, while HSCs and progenitor cells follow a completely opposite trend (Hirakawa et al., 2023). In A-ZIP/F-1 mice, hematopoietic recovery after irradiation is accompanied by a higher leukocyte count in peripheral blood and an increase in hematopoietic progenitors in bone marrow compared to WT mice, suggesting that BMAT depletion may facilitate hematopoietic reconstitution (Naveiras et al., 2009; Zhou et al., 2017). Pharmacological inhibition of adipocyte formation during hematopoietic recovery after irradiation further supports this notion, as it enhances bone marrow engraftment in WT mice (Naveiras et al., 2009). Taken together, these findings support a model in which BMAds act as negative regulators of hematopoisis.

Despite this evidence, a growing body of work has revealed that BMAds can also exert positive effects on hematopoiesis, through mechanisms that appear to differ between sites of red marrow (long bones) and those with yellow marrow (caudal vertebrae). A model with DTA-induced loss of BMAds (*BMAd-Cre;DTA*) shows that caudal vertebrae display reduced HSC and progenitor cell numbers and proliferative capacity (Li et al., 2022a). Similarly, BMAT-specific deletion of the stem cell factor gene (*Scf*, an important HSC niche factor) using an *Adipoq* promoter (*Adipoq-cre/ER;Scf^GFP/fl^* mice) reduces total bone marrow cellularity and HSC numbers in caudal vertebrae, but not in femurs and tibiae, which remain comparable to WT mice (Zhou et al., 2017). This site-specificity likely reflects the much lower BMAd content of femurs and tibiae relative to caudal vertebrae, and the fact that *Adipoq*-Cre may have targeted almost exclusively MALPs or *Adipoq*-expressing SSPCs at these red marrow sites (Palmisano et al., 2024). These data therefore identify SCF as an important BMAT-secreted niche factor whose influence scales with local adipose tissue content rather than skeletal site per se. Consistently, myeloablation – which markedly increases BMAT in femurs and tibiae – in *Adipoq-cre/ER;Scf^GFP/fl^* mice impairs hematopoiesis regeneration at these skeletal sites, further supporting this conclusion (Zhou et al., 2017).

The apparent incongruence between these results and those from A-ZIP/F1 mice, where hematopoisis regeneration after irradiation is increased in caudal vertebrae but reduced in BMAT-depleted long bones (Zhou et al., 2017) can be reconciled by considering the nature of the models. Genetic manipulation of a single molecule, such as *Scf*, selectively alters one BMAd function while leaving the rest of the tissue intact. Constitutive BMAT depletion, as in A-ZIP/F1 mice, instead removes the entire adipocyte compartment, creating a non-physiological condition with potentially profound and pleiotropic effects on the bone marrow microenvironment. BMAds are indeed never depleted in physiological conditions. They exhibit, contrarily, high plasticity: rather than simply accumulating or disappearing, BMAds expand during myeloablation and subsequently dedifferentiate into leptin receptor-expressing SSPCs during hematopoietic regeneration, a process driven by *Atgl*-dependent lipolysis (Hirakawa et al., 2023). Accordingly, BMAd-specific deletion of *Atgl* – using either a *FAC-Cre* (Li et al., 2022b) or a *Plin1*-CreER (Hirakawa et al., 2023) promoter – severely impairs HSC regeneration (Hirakawa et al., 2023; Li et al., 2022b), underscoring the functional importance of BMAd lipolysis and plasticity in supporting hematopoiesis.

The association between BMAT and hematopoiesis extends to human settings. *In vitro*, adipogenically differentiated human bone marrow stromal cells (BMSCs) support myeloid and erythroid differentiation of HSCs and progenitor cells (Boyd et al., 2017). *In vivo*, the relevance of this relationship is highlighted by acute myeloid leukemia (AML), in which myelo-erythroid populations are suppressed alongside a reduction in the number and size of BMAds (Boyd et al., 2017). Studies in human AML xenograft mouse models show that, upon engraftment, AML cells selectively reduce BMAT within red marrow regions containing rBMAds – such as femurs – while cBMAT remains unaffected (Boyd et al., 2017). Importantly, *in vivo* administration of a PPARγ agonist, which promotes adipogenesis and preserves BMAT, protects healthy myelo-erythropoiesis from leukemic suppression (Boyd et al., 2017), directly implicating BMAT in sustaining hematopoietic output within the bone marrow niche.

Altogether, these findings indicate that, despite the clear negative association between BMAd number and hematopoietic capacity, BMAT-related mechanisms regulating the HSC niche exist and are critically important for physiological hematopoiesis.

## Causal role in skeletal and non-skeletal disorders

### BMAT and osteoporosis

As one of the most frequent and clinically significant pathological conditions affecting bone, osteoporosis represents a major public health burden, often leading to fragility fractures and severe morbidity. For a long time, the increase in BMAT has been recognized as a hallmark of osteoporosis. BMAT expansion is indeed strongly correlated with bone loss, while its association with incident fractures is controversial (Schwartz et al., 2013; Woods et al., 2020; Xu et al., 2025b). This has been traditionally explained by a shift toward the adipogenic lineage, at the expenses of the osteogenic one, of SSCs. Estrogen may play a role in regulating SSCs (Palmisano et al., 2022; Wu et al., 2018); however, as BMAT is increased in female patients with post-menopausal osteoporosis but also in osteoporotic males, it suggests that other mechanisms are likely involved in the accumulation of fat in the bone marrow. Estrogens are also produced in men (Simpson et al., 1994), albeit at much lower levels than in women, and their bioavailability declines with age through the action of sex-hormone-binding globulin (SHBG), which binds estrogens and androgens and reduces their availability (Longcope et al., 2000). This mirrors what occurs in women, though to a much lesser degree; a regulatory role for estrogen in male BMAT accumulation therefore cannot be excluded. In support of this, recent large-scale genome-wide association studies have found that genes linked to altered bone marrow fat fraction are enriched for pathways in estrogen signaling (Xu et al., 2026).

While the hypothesis about SSC differentiation skewing still remains the most accepted to explain the reduced bone and increased BMAT in osteoporosis, recent evidence has also suggested a possible direct contribution of BMAds to the bone loss. The contribution of BMAds to osteoclastogenesis through RANKL expression has led to the hypothesis that BMAT expansion may be a causal event in the bone loss observed in osteoporosis. However, this has not been substantially proved, neither in humans nor in mice. Indeed, healthy males exhibit more BMAT than females at many skeletal sites – including the femoral head, total hip and femoral diaphysis (Xu et al., 2025a) – yet also display greater bone mass under physiological conditions. This dissociation argues against a straightforward causal role of BMAT in driving bone resorption. Furthermore, the possibility that osteoporosis itself drives BMAT expansion, and the consequent increase in RANKL release, cannot be excluded, making it difficult to establish the direction of causality. This hypothesis, however, struggles to account for the fact that BMAT expansion in postmenopausal women compared to age-matched males is restricted to the spine and does not extend to other skeletal sites. These considerations raise questions about the direct role of mature BMAds in regulating bone resorption through RANKL or M-CSF. As discussed in the previous paragraph, recent findings increasingly point to BMAd precursors, such as MALPs, as the primary stimulators of osteoclastogenesis, and these cells may play a more direct role in the pathophysiology of osteoporosis (Lu et al., 2025).

Instead, BMAd lipid metabolism may be a prominent regulator of osteoblast wellness. A recent study has shown that mouse BMAds during OVX-induced osteoporosis deteriorate the skeletal micro-environment by secreting large amounts of lipids, altering the senescence status of neighboring cells and affecting their mitochondrial function (Lin et al., 2025). Inhibition of lipid metabolism and secretion in BMAds, rescues osteoblasts from mitochondrial dysfunction-induced senescence and restores osteogenesis (Lin et al., 2025). Finally, recent studies using MRI data from patients in UK Biobank strengthened the evidence of causality between increased bone marrow fat fraction (BMFF, a measure of BMAT in MRI) and osteoporosis in humans, as mendelian randomization analyses revealed a causal link between femoral BMFF and osteoporosis (Ahmed et al., 2025; Wu et al., 2025; Xu et al., 2025b).

### BMAT and cancer

Bone marrow can be affected by hematological tumors and is one of the most frequent sites colonized by metastases, particularly in the case of breast, prostate and lung cancers. There is consistent evidence showing that BMAds can fuel cancer cells supporting their survival and proliferation promoting tumor growth and progression. On the other side, cancer cells are able to induce metabolic and molecular changes in BMAT that makes it produce and secrete factors (like adipokines and fatty acids) that create a favorable microenvironment for tumor survival and growth, acting as energy source for cancerous cells. Immunophenotypic analysis of human bone marrow samples affected by hematological and myeloproliferative tumors, and bone metastasis, showed a reduction in the number and size of BMAds (Dello Spedale Venti et al., 2022; Fairfield et al., 2021; Frączak et al., 2018; Heydt et al., 2021; Lu et al., 2018). These morphological changes can be associated with the expression of phospho-hormone-sensitive lipase (p-HSL), a lipase involved in the breakdown of triglycerides, suggesting an enhancement of lipolysis to fuel cancer cells (Dello Spedale Venti et al., 2022). Indeed, the smaller and lipolytic BMAds populating the bone marrow of acute myeloid leukemia (AML) patients are associated with a poor prognosis (Lu et al., 2018). Moreover, hematological tumor cells, such as in AML and multiple myeloma (MM), directly induce lipolysis and release of FFAs by BMAds, which are taken up by cancer cells through fatty acid transporter proteins (such as FABP4), to promote tumor proliferation (Panaroni et al., 2022; Shafat et al., 2017). Furthermore, BMAds and their precursors promote the migration of MM cells *in vitro* though the secretion of chemoattractant molecules such as monocyte chemotactic protein-1 (MCP-1) and stromal cell-derived factor-1α (SDF-1α) (Trotter et al., 2016). BMAd precursor cells can also influence MM cell migration and dissemination *in vivo*, since mice injected with MM cells after being co-cultured with preadipocytes show a higher tumor burden compared to those injected with MM cells cultured alone (Trotter et al., 2016). In agreement with this mechanistic data, recent phenome-wide association and mendelian randomization studies using MRI data from patients in UK Biobank identified a positive association between increased femoral diaphysis BMFF and MM in humans (Xu et al., 2025b), further supporting a role for BMAds in MM pathogenetic mechanisms.

Breast cancer cells migrate preferentially towards BMAds when colonizing bone marrow and get in close interactions with fat cells through pseudopodial extensions (Gaculenko et al., 2020; Templeton et al., 2015). In high-fat-diet (HFD) mice, showing an accumulation of BMAT, metastatic tumor progression is enhanced after intra-tibial melanoma cells injection (Gaculenko et al., 2020). A similar finding is also observed in HFD rats showing an increased metastatic tumor burden after intra-arterial human breast cancer cells injection (Gaculenko et al., 2020). Moreover, this increased metastatic tumor expansion in both HFD mice and rats is prevented by antagonizing adipocyte differentiation through treatment with the PPARγ inhibitor bisphenol-A-diglycidyl ether (BADGE), suggesting that the increased metastatic tumor burden in both HFD mice and rats is due to the higher amount of BMAT (Gaculenko et al., 2020).

A recent preprint article showed that rBMAds promote migration and invasiveness of metastatic prostate cancer cells inducing migration-related pathways and epithelial mesenchymal transition (EMT) program (Hernandez et al., 2025 preprint). This migratory phenotype induction in metastatic prostate cancer cells is mediated by rBMAds-released FFAs that are taken up by prostate cancer cells inducing a transcriptional reprogramming that promotes motility (Hernandez et al., 2025 preprint). Specifically, Angiopoietin-like 4 (ANGPTL4) is one of most overexpressed genes in prostate cancer cells induced by FFA stimulation (Hernandez et al., 2025 preprint). Furthermore, beside promoting proliferation and invasiveness of cancer cells, BMAds can also protect tumor cells against chemotherapy. In fact, human BMAds are able to protect MM cells from chemotherapy-induced apoptosis, by activating autophagy in cancer cells through the secretion of some adipokines such as leptin and adipsin (Liu et al., 2015). This effect is observed both *in vitro* and *in vivo*, since in mice injected with MM cells and BMAds directly into the femurs, and subjected to chemotherapy treatment, the tumor burden is higher than in mice injected with MM cells alone, as cancer cells are protected from chemotherapy-induced apoptosis by the BMAd-dependent autophagy activation (Liu et al., 2015). A similar role by BMAds in protecting cancer cells against chemotherapy is also observed for acute lymphoblastic leukemia (ALL) cells, since BMAds induce a slow-proliferation and cellular quiescence state in cancer cells inhibiting protein biosynthesis and can potentially lead to chemotherapy resistance (Heydt et al., 2021).

## Open questions and future perspectives

The past decade has transformed our understanding of BMAT from an inert space-filler to an active participant in skeletal and hematopoietic pathophysiology. Yet, fundamental questions remain unresolved, and several emerging areas promise to reshape the field in the years ahead. One of the most pressing unresolved issues is the precise causal relationship between BMAT expansion and bone loss. Although BMAT and bone mass are frequently inversely correlated, and Mendelian randomization studies now support a causal link between elevated bone marrow fat fraction and osteoporosis risk, the directionality and magnitude of this relationship vary considerably across skeletal sites, sexes, and disease contexts. Loss of BMAT does not uniformly translate into bone gain, and BMAT expansion does not invariably produce bone loss. Disentangling whether BMAT accumulation is a driver of skeletal deterioration, a compensatory response, or a passive reflection of SSPC differentiation bias remains a central challenge. Answering this question will require refined genetic tools that allow precise, inducible, and spatially restricted manipulation of BMAT *in vivo*, ideally distinguishing between rBMAT and cBMAT subtypes and between mature adipocytes and their precursor populations.

A second major frontier concerns the identity and heterogeneity of BMAd precursors. Despite significant advances in defining SSPC trajectories through single-cell transcriptomics and lineage tracing, the specific progenitor populations giving rise to rBMAds versus cBMAds have not been identified. The existence of regionally distinct precursors – as it seems to be the case for more osteochondrogenic SSCs (Ambrosi et al., 2025) – with different adipogenic thresholds and responses to niche signals, would explain much of the spatial variability in BMAT behavior but remains to be formally demonstrated. At this regard, the limitations of widely used *Cre* drivers, particularly *Adipoq-Cre*, which targets not only mature adipocytes and MALPs but also SSPCs with osteogenic potential, must be carefully addressed in experimental designs and interpretations.

The functional relationship between BMAT and the HSC niche represents another area of active investigation. Current evidence supports both positive and negative regulatory roles for BMAT in hematopoiesis, with the outcome depending on the specific molecular mechanisms engaged, the skeletal site, and the physiological state of the animal. BMAd plasticity (i.e. the capacity of mature adipocytes to dedifferentiate into leptin receptor-expressing SSPCs following myeloablation) adds a layer of complexity that is only beginning to be appreciated. Understanding the molecular signals that trigger and regulate this dedifferentiation program and determining its relevance in non-myeloablative conditions and in human physiology, represent important goals for the field. The discovery that BMAd-derived SCF is a critical niche factor for HSC maintenance in BMAT-rich skeletal sites raises the broader question of how many other secreted factors from BMAds or MALPs contribute to HSC homeostasis, and whether these can be therapeutically exploited to improve hematopoietic recovery after injury or transplantation.

In the oncological setting, the role of BMAT in supporting tumor cell survival, metabolic adaptation, and chemoresistance is increasingly well-documented, yet the therapeutic implications remain largely unexplored. The ability of BMAds to fuel cancer cells through lipolysis-driven fatty acid transfer, to activate autophagy and quiescence programs that shelter leukemic cells from cytotoxic therapy, and to remodel the marrow niche in favor of malignant expansion, all point to BMAT as a potential therapeutic target in hematological malignancies and bone metastatic cancers. Pharmacological strategies that could modulate BMAT, such as PPARγ agonists to preserve healthy adipogenesis in AML, or lipid metabolism inhibitors to deprive tumor cells of energy substrates, deserve investigation.

Finally, the contribution of BMAT to systemic metabolism and immunology, a topic that was not discussed in this Review, deserves greater attention. Aging-associated BMAT expansion coincides with increased systemic inflammation and immune senescence, and BMAds may contribute to this phenotype through the secretion of pro-inflammatory adipokines and lipid mediators. Whether BMAT participates in the regulation of systemic glucose homeostasis, as it is suggested by its distinct glucose uptake profile, remain open questions with broad implications.

In summary, BMAT is no longer ‘just a filler’. It is a multifunctional tissue whose dissection at molecular, cellular, and organism levels promises to unlock new insights into aging, skeletal disease, hematological disorders, and cancer. The convergence of advanced genetic tools, single-cell technologies, human genomics, and clinical imaging now provides an unprecedented opportunity to elevate BMAT from a bone marrow curiosity to a bona fide therapeutic target.
